# Milk: the new sports drink? A Review

**DOI:** 10.1186/1550-2783-5-15

**Published:** 2008-10-02

**Authors:** Brian D Roy

**Affiliations:** 1 Centre for Muscle Metabolism and Biophysics, Faculty of Applied Health Sciences, Brock University, St. Catharines, Ontario, Canada

## Abstract

There has been growing interest in the potential use of bovine milk as an exercise beverage, especially during recovery from resistance training and endurance sports. Based on the limited research, milk appears to be an effective post-resistance exercise beverage that results in favourable acute alterations in protein metabolism. Milk consumption acutely increases muscle protein synthesis, leading to an improved net muscle protein balance. Furthermore, when post-exercise milk consumption is combined with resistance training (12 weeks minimum), greater increases in muscle hypertrophy and lean mass have been observed. Although research with milk is limited, there is some evidence to suggest that milk may be an effective post-exercise beverage for endurance activities. Low-fat milk has been shown to be as effective, if not more effective, than commercially available sports drinks as a rehydration beverage. Milk represents a more nutrient dense beverage choice for individuals who partake in strength and endurance activities, compared to traditional sports drinks. Bovine low-fat fluid milk is a safe and effective post exercise beverage for most individuals, except for those who are lactose intolerant. Further research is warranted to better delineate the possible applications and efficacy of bovine milk in the field of sports nutrition.

## Background

Nutritional intake is important for optimizing sport and exercise performance. Furthermore good nutrition is important in optimizing adaptations to training. For example, the ancient Greeks believed that high protein intakes were important for athletes, and these athletes would consume diets that contained excessive amounts of meat. Such ideas are still pervasive today, especially with resistance based sports such as body building. It is common for resistance athletes to consume diets that are more than double the recommended levels of dietary protein. In addition, athletes who participate in body building and similar sports are bombarded with marketing for various supplements, many of which are very high in protein. Research has clearly demonstrated that such excessively high protein intakes aren't necessary to facilitate the adaptations that occur with resistance training. Research has also established that the timing of nutritional intake is also very important in optimizing the adaptations to this form of exercise, as well as recovery from both resistance and endurance exercise. Finally, the nutrient composition of post-exercise nutritional intake has also been shown to be important in the recovery from endurance exercise and adaptations/recovery from resistance exercise.

Bovine based milk and milk products represent a very good source of proteins, lipids, amino acids, vitamins and minerals. The health benefits of milk have been well established and have been extensively reviewed elsewhere [1]. Low-fat milk has a number of characteristics that theoretically make it a potentially good recovery beverage (Table 1). Firstly, it contains carbohydrates (lactose) in amounts similar to many commercially available sports drinks (glucose, maltodextrin). Milk contains casein and whey proteins in a ratio of 3:1 which provides for slower digestion and absorption of these proteins resulting in sustained elevations of blood amino acid concentrations [2]. Another advantage is that whey protein also contains a large proportion of branched chain amino acids which have an integral role in muscle metabolism and protein synthesis. Finally, milk also has naturally high concentrations of electrolytes, which are naturally lost through sweating during exercise. The high concentrations of these electrolytes should aid in fluid recovery following exercise. Based on these characteristics of milk, there has been growing sport nutrition research interest in milk and its possible roles as an exercise beverage for both resistance and endurance sports and training.

**Table 1:** Nutrient Information for milk and various sports drinks

	**Whole Milk (3.25%)**	**Partly Skimmed (2%)**	**Partly Skimmed (1%)**	**Skim Milk (0.1%)**	Chocolate Milk, Partly Skimmed* (2%)	**Gatorade Thirst Quencher**^**®**^	**Gatorade Endurance Formula**^**®**^	**Accelerade**^**®**^
**Kcal**	159	128	108	90	189	52	53	85
**kJ**	663	536	453	380	793	218	221	354
**Protein (g)**	9	9	9	9	8	0	0	4
**Fat (g)**	9	5	3	trace	5	0	0	0
**Carbohydrate (g)**	12	12	12	13	27	15	15	16
**Sodium (mg)**	126	129	129	133	159	115	211	127
**Potassium (mg)**	391	398	402	431	446	31	95	16

All presented information is based on a 250 mL serving of each of the different beverages. Data is from , , and . *Chocolate milk has added sucrose and cocoa and the macronutrient composition of chocolate milk varies depending on the specific manufacturer.

## Milk and resistance exercise and training

Resistance exercise and resistance sports are characterized by repeated high intensity contractions of varying muscle groups that leads to well characterized adaptations in muscles [3]. The most obvious adaptation is skeletal muscle hypertrophy. For muscle hypertrophy to occur there must be a chronic increase in muscle protein net balance. Muscle protein balance is a function of muscle protein synthesis and muscle protein breakdown. Thus, for an increase in net balance to occur, there must be an increase in protein synthesis, a decrease in muscle protein breakdown or simultaneous increase in synthesis and decrease in breakdown. Over the last decade there has been considerable investigation into the influence of various factors that influence the protein metabolism response to resistance exercise.

Resistance exercise alone results in both an increase in protein synthesis and protein breakdown, but the increase in synthesis is greater than the increase in breakdown, resulting in a less negative net balance [4]. Interestingly, the results from Phillips et al. did show a less negative protein balance, but the net balance was still negative since participants were in a fasted state [4]. These observations emphasised the importance of supplying macronutrients for influencing protein metabolism following exercise.

Several studies have documented the provision of macronutrients soon after resistance exercise in an attempt to optimize the protein metabolic response. Intake of amino acids [5], protein [6], carbohydrates [7-9], or mixed macronutrient compounds [9-11] contribute to enhanced protein metabolism following resistance exercise. This work has documented that the protein-related metabolic response following resistance exercise can be influenced through the nutritional intake of the main macronutrient constituents of low-fat milk; protein and carbohydrates. Follow-up studies have directly investigated the impact of milk consumption on the acute protein metabolic response following resistance exercise.

Elliot et al. investigated the influence of consuming differing milk beverages on the protein metabolic response following an acute bout of resistance exercise [12]. They compared the influence of non-fat milk (237 g), whole milk (237 g) and an amount of non-fat milk with the same amount of energy (kJ) as the whole milk condition (393 g) following a bout of leg resistance exercise. They assessed amino acid net balance across the exercise leg for 5 hours following the leg resistance exercise. All of the different milk beverages resulted in a significant increase in net balance of the measured amino acids. This study did not determine what contributed to the change in net balance (change in synthesis, change in breakdown, or both), however, the evidence did show that protein metabolism was enhanced with a single bolus intake of milk after the resistance exercise.

Recent work has shown that one way by which consumption of fat-free milk increase protein net balance is through an increased rate of muscle protein synthesis following resistance exercise [13]. The increase in protein net balance and muscle protein synthesis was most pronounced with the consumption of 500 mL of fat-free milk as compared to an isoenergetic, isonitrogenous, and macronutrient matched soy-protein beverage (745 Kj, 18.2 g protein, 1.5 g fat, and 23 g carbohydrate). The authors speculated that their observations were attributable to the differences in soy protein digestion as compared to milk protein digestion. The soy based beverage was digested and absorbed much more rapidly leading to a large rapid rise in blood concentrations of amino acids shuttling them to plasma protein and urea synthesis [2], whereas with the fat-free milk the elevation in blood amino acids was slower and remained elevated for a more prolonged period, providing a more sustained delivery of amino acids for skeletal muscle protein synthesis.

Therefore, it is reasonable to hypothesize that milk beverages, when consumed soon after resistance exercise, can lead to enhanced improvements in protein metabolism following resistance exercise. Such acute increases in protein net balance and synthesis could possibly enhance the more chronic adaptations that occur with resistance training. Based on the acute changes in protein metabolism with milk consumption following resistance exercise, a number of questions arise. Firstly, what would be the application of this information over the long term and secondly, how can this be applied to athletes who are training on a regular basis. Recent research has addressed the long term influence of milk consumption after resistance training.

The first study to investigate the interaction of resistance training and milk consumption over a more prolonged period compared the effects of 3 days per week (for 10 weeks) of progressive resistance training with consumption of either low-fat chocolate milk (5 kcal/kg body weight) (composition described in table 1) or a commercially available carbohydrate and electrolyte beverage (5 kcal/kg body weight) within 5 min of completing each workout [14]. The post-exercise beverages contained the same amount of energy, but varied in macronutrient composition (Table 2). The training resulted in improvements in strength and body composition, however all of these changes where similar between the two groups. Essentially, no differences were observed for any of the measured variables when the group that consumed milk post exercise was compared to the group that consumed the commercial sports drink. However, there was an interesting trend for the milk group to have a greater increase in fat-free soft tissue (FFST) mass. The milk group gained 1.6 ± 0.4 kg of FFST mass, while the carbohydrate electrolyte beverage group only gained 0.8 ± 0.5 kg of FFST mass. These observations were not significantly different, but it would have been interesting to observe what would have occurred if the protocol had have been extend to see if these differences would have been more pronounced. Nonetheless, this was the first study to look at the long term interaction of milk consumption and resistance training.

**Table 2:** Macronutrient composition of supplements used by Rankin et al. 2004

	**CHO group**	**MILK group**
**Energy (Kcal/kg)**	5	5
**Carbohydrate (g/kg)**	1.25	0.92
**Protein (g/kg)**	0	0.21
**Fat (g/kg)**	0	0.06

Adapted from Rankin et al. 2004

More recently, Hartman et al. compared the consumption of three different post resistance exercise beverages during an extended period of intense resistance training in novice weightlifters [15]. The three different beverage conditions were; 1) fat-free milk (500 mL), 2) a soy beverage (500 mL) that was isoenergetic, isonitrogenous and macronutrient ratio matched to the fat-free milk, and 3) a control beverage (500 mL) that consisted of a flavoured carbohydrate beverage that contained maltodextrin [15]. The participants were randomized into the three different groups and trained 5 days per week for 12 weeks and consumed the appropriate beverage immediately and one hour following completion of each training session. Interestingly, consumption of the fat-free milk resulted in greatest increases in muscle hypertrophy, as observed through greater increases in both type I and II muscle fiber areas [15]. They also observed that the group that consumed the milk also gained the most lean body mass over the duration of the study [15]. Fat mass also declined to the greatest extent in the milk group [15]. The authors attributed the greater increase in muscle fiber hypertrophy and lean mass to the previously observed acute influences of milk consumption on protein metabolism [13]. The greater decline in fat mass observed with the milk group was suggested to be related to the greater calcium intake associated with the milk consumption [15], as there is growing evidence to suggest a pivotal role for dairy products in influencing adipocyte metabolism in a manner that attenuates lipid accretion [16]. This very well controlled study clearly showed multiple benefits to using fat-free milk as a post-resistance exercise beverage.

There is growing amounts of evidence, both acute and long-term, to support the use of low-fat milk as a post-resistance exercise beverage. Consumption of low-fat milk appears to create an anabolic environment following resistance exercise and over the long term with training, it appears that greater gains in lean mass and muscle hypertrophy can be obtained [15]. Furthermore, milk may also lead to greater losses of body fat when it is consumed following resistance training [15].

## Milk and endurance exercise and training

Endurance sports and activities are generally considered to be sub-maximal activities that can be performed for more prolonged periods of time. These activities are also characterized by continuous exercise/activity that is highly dependent on oxidative metabolism as a source of energy and usually involve large muscle groups. This dependence on oxidative metabolism and involvement of a large muscle mass leads to higher rates of total substrate turnover and under certain conditions, depletion of muscle glycogen in the active muscles. However, there can be considerable differences in the responses observed depending on the intensity of the exercise performed.

From a nutritional standpoint, there are three primary times when nutrition is considered for endurance based activities; before exercise, during exercise, and after exercise. Each time period of nutritional concern has varying objectives. Prior to exercise, nutritional goals are to ensure that the athlete is well fuelled and that any nutritional intake will not interfere with the normal physiological responses to the activity. The goal of nutritional intake during exercise is to provide exogenous substrates in an attempt to delay the depletion of endogenous substrates, and to provide fluids to offset fluid losses due to sweating. Finally, the nutritional objectives of post-exercise nutrition are to promote muscle recovery and adaptations, fuel resynthesis in muscle, and fluid replenishment. With specific regard for the role of milk as a nutritional option for endurance activities, there is limited research into the possible benefits of milk and it is also difficult to extend findings into any consistent recommendations because of differences in design and methodology. However, a number of recent studies suggest that there is significant potential for expanded research in this area, especially in the area of recovery from endurance exercise.

What is evident is that when milk is compared to carbohydrate based sports drinks, similar responses in many physiological variables are observed during the exercise. There have been some minor differences reported such as increased concentrations of essential amino acids [17], based on the protein content of milk. Interestingly, when milk is consumed during prolonged exercise, following completion of the exercise a reduction in whole body protein breakdown and protein synthesis was observed with a simultaneous increase in protein oxidation [18]. The authors speculated that the decrease in whole body protein synthesis might have been due to preferential oxidation of the ingested protein during the exercise, leaving less amino acids available for synthesis after the exercise [18]. Another difference that has been reported is that participants reported greater feelings of stomach fullness with milk as compared to water or carbohydrate based beverages [19]. The increase in stomach fullness possibly suggests that the rate of fluid intake may have been greater than gastric emptying [19], which would not have been unexpected as the rate of gastric emptying declines with increasing energy density of the fluid consumed [20]. Despite these reported variations in physiological responses and stomach fullness, there were no reported differences in actual performance measures. For example, when participants rode at a set intensity until exhaustion, milk and carbohydrate based sports drinks resulted in similar times to exhaustion, suggesting that milk is just as beneficial as commercially available sports drinks at delaying the onset of fatigue under these conditions [19]. Clearly, additional research is required to further establish the efficacy of milk as an endurance exercise supplement beverage. Future research should include time trial based performance measures, as they are more realistic to endurance sport performance. Furthermore, future research should also strive to develop a greater understanding of the metabolic influence of milk consumption during prolonged exercise, as there is very limited research in this area.

The use of milk as a recovery beverage after endurance exercise has also been investigated to a limited extent. The main goal of any post exercise nutritional intervention is usually to promote muscle glycogen resynthesis, and fluid recovery. In regards to glycogen resynthesis, there is very limited direct research into the efficacy of milk consumption to replenish muscle glycogen levels. However, there is some performance based data that suggests that chocolate milk is as effective as a commercially available sports drink in facilitating recovery [21] (See Table 3 for drink composition). A group of trained endurance athletes were used to compare the effectiveness of varying endurance recovery beverages, one being chocolate milk, following a series of glycogen depleting intervals [21]. The chocolate milk drink and the carbohydrate recovery drink were controlled for based on carbohydrate and energy content. After four hours of recovery and consumption of the different beverages, the participants performed a ride to exhaustion. The time to exhaustion and total work performed during the performance trial was the same when the participants consumed chocolate milk or a common commercially available sports drink [21]. Based on these findings one could speculate that chocolate milk may be as effective as more commonly used sports drinks at promoting glycogen resynthesis. However, to date there have been no well controlled studies that have directly measured the efficacy of milk to promote muscle glycogen recovery following prolonged endurance exercise. Therefore, future research should directly quantify rates of muscle glycogen resynthesis following prolonged endurance exercise and compare the efficacy of milk to other commonly used recovery beverages.

**Table 3:** Composition of chocolate milk drink used by Karp et al. 2006

	**Chocolate milk drink**	**CHO recovery drink**
**Kcal**	187.5	187.5
**kJ**	787.1	787.1
**Protein (g)**	9.4	9.1
**Fat (g)**	2.6	0.7
**Carbohydrate (g)**	34.4	34.4
**Sodium (mg)**	197.9	152.8
**Potassium (mg)**	443.8	83.3

Adapted from Karp et al. 2006. All values are based on 250 mL of each drink.

The other previously stated goal of a post endurance exercise beverage is to promote rehydration due to the excessive fluid loss that occurs as a result of sweating. To date there has been one well controlled study that investigated the effectiveness of low-fat milk as a rehydration beverage [22]. In this study they compared the effectiveness of low-fat milk alone, low-fat milk with additional sodium chloride, a sports drink, and water at restoring fluid balance after exercise in a hot environment (1.8% loss in body mass). The volume of each drink consumed was 150% of the volume of fluid lost during the exercise. The intake of 150% of fluid lost is a common recommendation for rehydration after exercise. The different recovery beverages were separated into four equal amounts and were given to the participants every 15 minutes during the recovery period. Urine output and net fluid balance was determined during 4 hours of recovery. Urine output increased during the first two hours of recovery in all groups but the increase was attenuated in the two milk groups [22]. Furthermore, by the end of the 4 hours of recovery both milk groups were in a net positive fluid balance, while both the sports drink and water conditions remained in a net negative fluid balance [22]. The authors concluded that low-fat milk was an effective beverage for promoting rehydration following exercise induced dehydration, and that the low-fat milk was superior to a commercially available sports drink in promoting rehydration due to lower total urine output during recovery.

The ability of milk to effectively act as a rehydration beverage likely relates to the composition of milk. Milk naturally has high concentrations of electrolytes (133 mg Na^+ ^and 431 mg K^+ ^in a 250 mL serving) which aid in fluid retention when consumed. Another factor that has been speculated to contribute to the ability of milk to be an effective post-exercise rehydration beverage is the rate at which it empties from the stomach [22]. Energy dense fluids empty from the stomach much more slowly, leading to a slower absorption into the circulation [23]. This slower absorption attenuates the large fluctuations in plasma osmolality that can occur with consumption of large volumes of water or sports drinks. Subsequently, the large fluctuations in osmolality (decreased osmolality) would result in increased clearance rates by the kidneys, similar to those observed by Shirreffs et al [22], resulting in large increases in urine output.

In summary, milk shows great promise as an alternative endurance exercise recovery beverage. The limited literature that does exist suggests that milk is as effective as commercially available sports drinks at facilitating recovery for additional performance, suggesting that it may be an effective beverage for promoting glycogen recovery. Furthermore, milk is also a very effective beverage at promoting fluid recovery following dehydrating exercise in the heat. More research is need to better understand how milk promotes recovery after exercise and to better understand the physiological mechanisms through which it acts.

## Conclusion

There is growing scientific evidence to support the use of low-fat milk following exercise by both individuals and athletes who habitually undertake strength or endurance training. There is data which suggests that fat free milk is as effective as, and possibly even more effective than, commercially available sports drinks at promoting recovery from strength and endurance exercise. Further work is required to better understand the physiological mechanisms by which milk exerts its actions following exercise and training. Milk also has the added benefit of providing additional nutrients and vitamins that are not present in commercial sports drinks. In conclusion, fat free milk is a safe and effective post-exercise beverage that has been shown to promote recovery from exercise and should be considered as a viable alternative to commercial sports drinks by lactose tolerant individuals.

## Competing interests

The author was invited to give a presentation on this topic at the 10^th ^Pan-American Dairy Congress in San Jose, Costa Rica, April 8^th ^to the 10^th^, 2008.

## References

[B1] Haug A, Hostmark AT, Harstad OM (2007). Bovine milk in human nutrition – a review. Lipids Health Dis.

[B2] Bos C, Metges CC, Gaudichon C, Petzke KJ, Pueyo ME, Morens C, Everwand J, Benamouzig R, Tome D (2003). Postprandial kinetics of dietary amino acids are the main determinant of their metabolism after soy or milk protein ingestion in humans. J Nutr.

[B3] Kraemer WJ, Duncan ND, Volek JS (1998). Resistance training and elite athletes: adaptations and program considerations. J Orthop Sports Phys Ther.

[B4] Phillips SM, Tipton KD, Aarsland A, Wolf SE, Wolfe RR (1997). Mixed muscle protein synthesis and breakdown after resistance exercise in humans. Am J Physiol.

[B5] Tipton KD, Ferrando AA, Phillips SM, Doyle DJ, Wolfe RR (1999). Postexercise net protein synthesis in human muscle from orally administered amino acids. Am J Physiol.

[B6] Tipton KD, Elliott TA, Cree MG, Wolf SE, Sanford AP, Wolfe RR (2004). Ingestion of casein and whey proteins result in muscle anabolism after resistance exercise. Med Sci Sports Exerc.

[B7] Borsheim E, Cree MG, Tipton KD, Elliott TA, Aarsland A, Wolfe RR (2004). Effect of carbohydrate intake on net muscle protein synthesis during recovery from resistance exercise. J Appl Physiol.

[B8] Roy BD, Tarnopolsky MA, MacDougall JD, Fowles J, Yarasheski KE (1997). Effect of glucose supplement timing on protein metabolism after resistance training. J Appl Physiol.

[B9] Roy BD, Fowles J, Hill R, Tarnopolsky MA (2000). Macronutrient intake and whole body protein metabolism following resistance exercise. Med Sci Sports Exerc.

[B10] Rasmussen BB, Tipton KD, Miller SL, Wolf SE, Wolfe RR (2000). An oral essential amino acid-carbohydrate supplement enhances muscle protein anabolism after resistance exercise. J Appl Physiol.

[B11] Tipton KD, Rasmussen BB, Miller SL, Wolf SE, Owens-Stovall SK, Petrini BE, Wolfe RR (2001). Timing of amino acid-carbohydrate ingestion alters anabolic response of muscle to resistance exercise. Am J Physiol Endocrinol Metab.

[B12] Elliot TA, Cree MG, Sanford AP, Wolfe RR, Tipton KD (2006). Milk ingestion stimulates net muscle protein synthesis following resistance exercise. Med Sci Sports Exerc.

[B13] Wilkinson SB, Tarnopolsky MA, Macdonald MJ, Macdonald JR, Armstrong D, Phillips SM (2007). Consumption of fluid skim milk promotes greater muscle protein accretion after resistance exercise than does consumption of an isonitrogenous and isoenergetic soy-protein beverage. Am J Clin Nutr.

[B14] Rankin JW, Goldman LP, Puglisi MJ, Nickols-Richardson SM, Earthman CP, Gwazdauskas FC (2004). Effect of post-exercise supplement consumption on adaptations to resistance training. J Am Coll Nutr.

[B15] Hartman JW, Tang JE, Wilkinson SB, Tarnopolsky MA, Lawrence RL, Fullerton AV, Phillips SM (2007). Consumption of fat-free fluid milk after resistance exercise promotes greater lean mass accretion than does consumption of soy or carbohydrate in young, novice, male weightlifters. Am J Clin Nutr.

[B16] Zemel MB (2004). Role of calcium and dairy products in energy partitioning and weight management. Am J Clin Nutr.

[B17] Miller SL, Maresh CM, Armstrong LE, Ebbeling CB, Lennon S, Rodriguez NR (2002). Metabolic response to provision of mixed protein-carbohydrate supplementation during endurance exercise. Int J Sport Nutr Exerc Metab.

[B18] Miller SL, Gaine PC, Maresh CM, Armstrong LE, Ebbeling CB, Lamont LS, Rodriguez NR (2007). The effects of nutritional supplementation throughout an endurance run on leucine kinetics during recovery. Int J Sport Nutr Exerc Metab.

[B19] Lee JK, Maughan RJ, Shirreffs SM, Watson P (2008). Effects of milk ingestion on prolonged exercise capacity in young, healthy men. Nutrition.

[B20] McHugh PR, Moran TH (1979). Calories and gastric emptying: a regulatory capacity with implications for feeding. Am J Physiol.

[B21] Karp JR, Johnston JD, Tecklenburg S, Mickleborough TD, Fly AD, Stager JM (2006). Chocolate milk as a post-exercise recovery aid. Int J Sport Nutr Exerc Metab.

[B22] Shirreffs SM, Watson P, Maughan RJ (2007). Milk as an effective post-exercise rehydration drink. Br J Nutr.

[B23] Maughan RJ, Leiper JB, Vist GE (2004). Gastric emptying and fluid availability after ingestion of glucose and soy protein hydrolysate solutions in man. Exp Physiol.

